# Neurokinin-1 receptor is a novel positive regulator of Wnt/β-catenin signaling in melanogenesis

**DOI:** 10.18632/oncotarget.13222

**Published:** 2016-11-08

**Authors:** Jia Zhou, Jingjing Ling, Huizhu Song, Bei Lv, Lei Wang, Jing Shang, Yong Wang, Chunyan Chang, Fengfeng Ping, Jun Qian

**Affiliations:** ^1^ State Key Laboratory of Natural Medicines, China Pharmaceutical University, Nanjing 210009, P.R. China; ^2^ Wuxi People's Hospital affiliated to Nanjing Medical University, Wuxi 214023, P.R. China

**Keywords:** neurokinin-1 receptor, Wnt/β-catenin, [Sar^9^, Met(O_2_)^11^] substance P, L-733060

## Abstract

Wnt/β-catenin signaling is essential for melanogenesis in melanocytes. Neurokinin-1 receptor (NK-1R) has recently been demonstrated to be involved in melanin production. However, the cross talk between NK-1R and Wnt/β-catenin is poorly understood. Here, [Sar^9^, Met(O_2_)^11^] substance P (SMSP) was used to activate NK-1R, while L-733060 was used to inhibit it. The effects of NK-1R activation and inhibition on Wnt and its inhibitors were analyzed using western blot and real-time quantitative PCR. The results showed that SMSP positively regulated Wnt/β-catenin signaling by increasing the expression of β-catenin and p-GSK3β protein, which resulted from the weakened expression of the Wnt inhibitor Dickkopf-1 (DKK1). On the contrary, L-733060 lowered the expression of β-catenin and p-GSK3β protein through the up-regulation of DKK1 expression. Furthermore, in L-733060-treated mice, it was found that the pigmentation level as well as the melanogenic proteins and β-catenin protein expression were down-regulated, while the expression of DKK1 was up-regulated. These results showed the interaction between NK-1R and Wnt in human melanocytes *in vitro* and C57BL/6J mice *in vivo*, indicating that NK-1R may positively regulate melanogenesis through Wnt/β-catenin signaling pathway.

## INTRODUCTION

Melanocytes are located in the basal layer of the epidermis and are responsible for producing melanin, a substance that gives skin and hair their pigments [1]. Melanogenesis is promoted by various stimulators such as UV irradiation, cytokines, growth factors, and hormones [2]. However, abnormal melanogenesis causes pigmentary disorders, including medical conditions such as hypopigmentation (vitiligo and albinism) or hyperpigmentation (solar lentigo, chlosma, and freckles) [3]. Wnt/β-catenin signaling is involved in many biological functions, such as melanogenesis [4, 5], neurogenesis [6, 7], and cancer initiation and progression [8, 9], and is triggered via binding of secreted Wnt proteins to Frizzled/low-density lipoprotein receptor-related protein complexes [10]. In the absence of Wnt, β-catenin is degraded by a multiprotein complex containing Disheveled (Dvl), GSK-3β, Axin, adenomatous polyposis coli (APC), and casein kinase1α (CK1α), which phosphorylates β-catenin and leads to its ubiquitination and proteasomal degradation [11, 12]. In the presence of Wnt, GSK-3β-dependent phosphorylation of β-catenin is blocked and β-catenin is translocated into the nucleus [13], which upregulates the expression of the microphtalmia-associated transcription factor (MITF) and recruits the complex of β-catenin and TCF/LEF to the binding sites of the MITF promoter [14]. The expression of Dickkopf-1 (DKK1) as an inhibitor of canonical Wnt/β-catenin signaling is responsible for pigmentation inhibition of melanocytes through suppression of β-catenin and MITF expression [15, 16].

Neurokinin, also called tachykinin, has been divided into three types including neurokinin-1 receptor (NK-1R), neurokinin-2 receptor (NK-2R) and neurokinin-3 receptor (NK-3R). They are G protein-coupled receptors encoded by three distinct genes [17, 18]. Substance P (SP), hemokinins and endokinins A and B bind with high affinity to NK-1R [19]. NK-1R is expressed on several cell types, including neurons, epithelial cells, adipocytes, and immuns cells [20, 21]. We have recently demonstrated the presence of NK-1R in the B16 melanoma cells and human primary melanocytes, along with the ability of SP to inhibit melanogenesis through the activation of NK-1R [22]. Considering the involvement of NK-1R and Wnt/β-catenin in melanogenesis, it is implicated that there may exist a certain relationship between NK-1R and Wnt/β-catenin.

**Figure 1:**
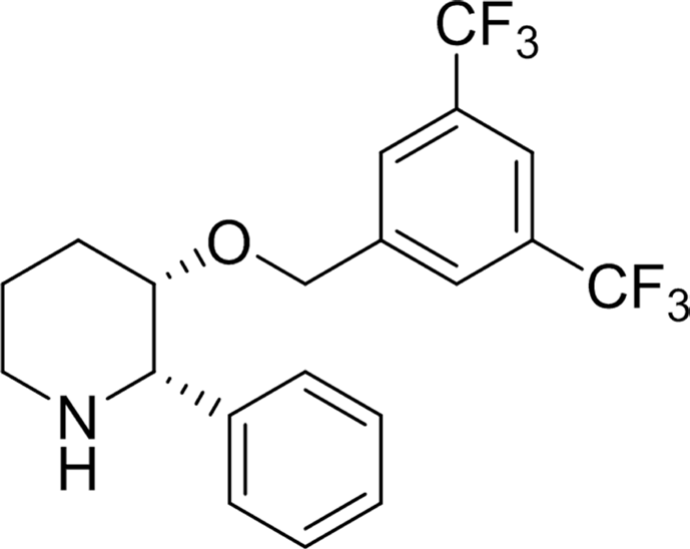
Chemical structures of (2S,3S)-3-[(3,5-bis (Trifluoromethyl) phenyl) methoxy]-2-phenylpiperidine hydrochloride (L-733060)

L-733,060 (Figure 1) is a selective, potent, and long-acting central nonpeptide tachykinin NK1 receptor antagonist showing high affinity for the human NK1 receptor *in vitro* [23]. It has also been demonstrated that L-733,060 exerts antitumor activity against human melanoma, neuroblastoma, glioma, retinoblastoma, pancreas, larynx, gastric and colon carcinoma cell lines [24–30]. However, to our knowledge, no study has been performed on the effect of L-733,060 on Wnt/β-catenin. Thus, the purpose of this study was to demonstrate, using a NK1 receptor antagonist L-733060 and a NK1 receptor agonist SMSP, to evaluate the effects of NK-1R on Wnt/β-catenin in the human primary melanocytes and C57BL/6J mice.

## RESULTS

### NK-1R activation promotes Wnt/β-catenin signaling pathway in human melanocytes

**Figure 2:**
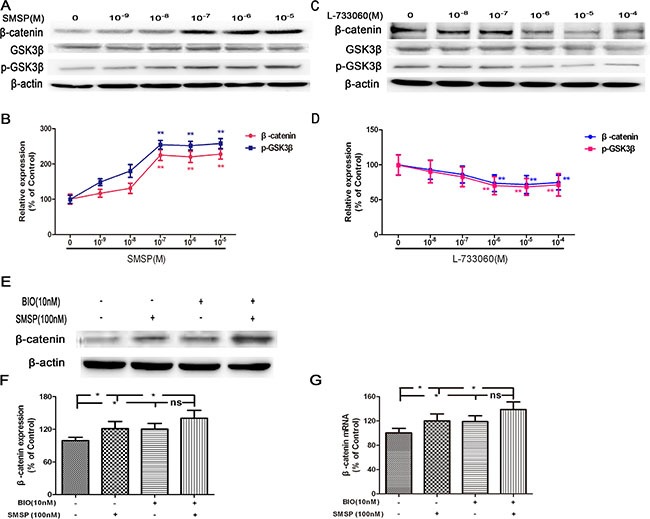
The effects of NK-1R activation (using SMSP) and inhibition (using L-733060) on p-GSK3β, GSK3β and β-catenin protein expression (**A**–**B**) SMSP (at 10^−9^–10^−5^M) could enhance p-GSK3β and β-catenin protein expression. (**C**–**D**) L-733060 (at 10^−8^–10^−4^ M) could inhibit p-GSK3β and β-catenin protein expression. After treatment with BIO, the expression of β-catenin was detected by Western blot (**E**–**F**) and Real-time qRCR (**G**). Statistical results from the densitometric measurements after normalization against β-actin were calculated as the mean ± SD (*n* = 6). Values are expressed as a percentage of the corresponding control value. ^**^p < 0.01, ^*^p < 0.05.

To analyze the relevance between NK-1R and Wnt/β-catenin in melanogenesis, we examined whether NK-1R directly regulates the expression of β-catenin and GSK3β, the key genes and proteins implicated in the Wnt/β-catenin signaling pathway. In western blot experiments, we found that SMSP (at 10^−9^–10^−5^ M) increased the expression of β-catenin and phosphorylated GSK3β (p-GSK3β) (Figure 2A and 2B), while the protein expression level of GSK3β was unchanged. On the contrary, L-733060 (at 10^−8^–10^−4^M) treatment decreased β-catenin and p-GSK3β expression (Figure 2C and 2D) but not GSK3β.

Next, to examine whether the expression level of β-catenin protein is directly regulated by GSK-3β, we treated melanocytes with a GSK3β inhibitor BIO, a highly selective inhibitor for 72 h before SMSP treatment. As shown in Figure 2E–2G, pretreatment with BIO could decrease the activity of GSK3β and further promote the up-regulation of β-catenin induced by SMSP. These findings indicate that NK-1R is a positive regulator of Wnt/β-catenin signaling pathway in melanocytes.

### NK-1R activation decreases DKK1 expression in human melanocytes

**Figure 3:**
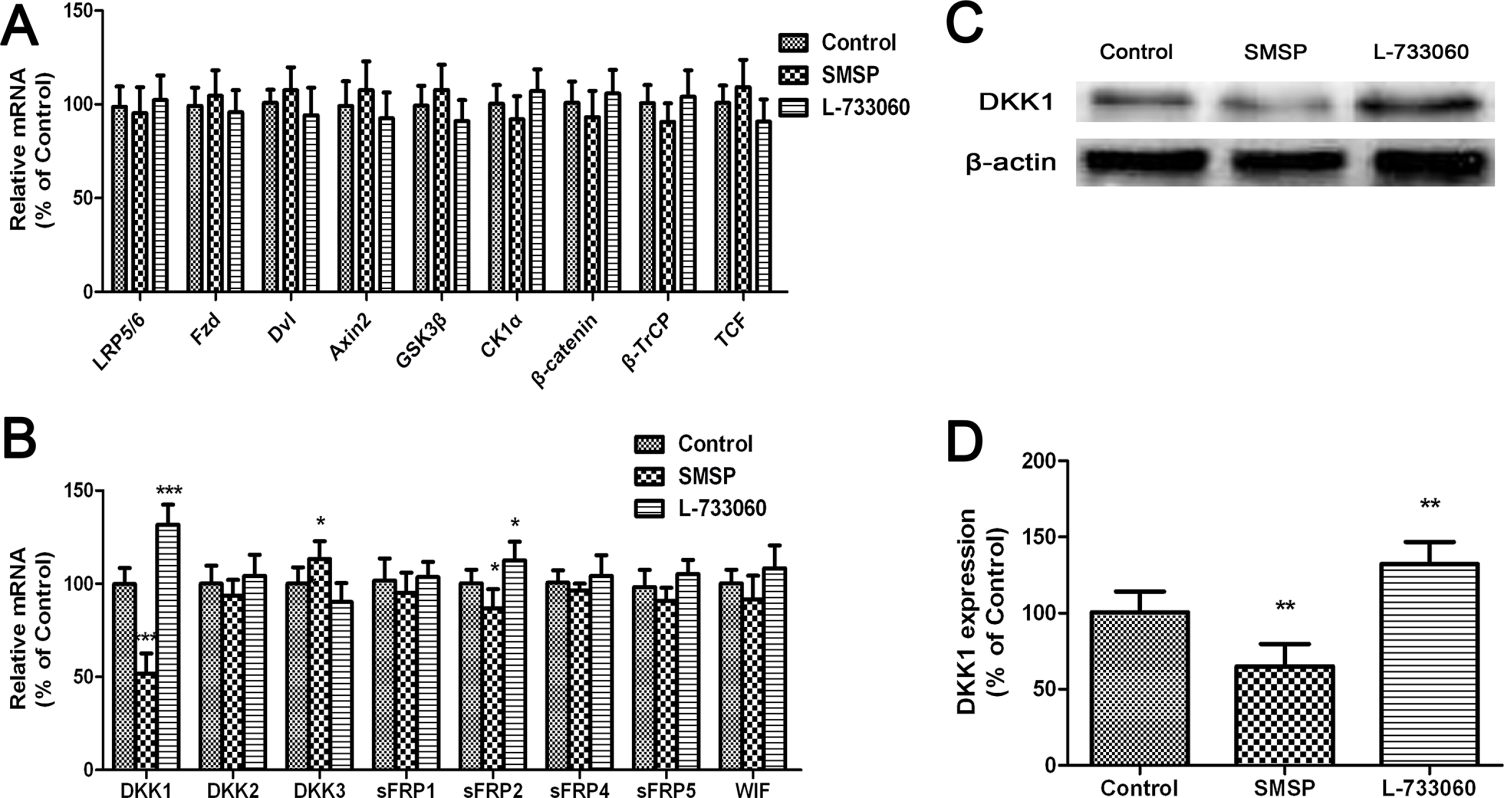
The effects of NK-1R activation (using SMSP) and inhibition (using L-733060) on Wnt inhibitor expression (**A**) mRNA levels of receptors, ligands, and many intracellular factors involved in the Wnt/β-catenin signaling pathway were analyzed by quantitative real-time PCR. (**B**) mRNA levels of Wnt inhibitors were analyzed by quantitative real-time PCR. (**C**–**D**) protein levels of DKK1 were analyzed by western blot. Statistical results from the densitometric measurements after normalization against β-actin were calculated as the mean ± SD (*n* = 6). Values are expressed as a percentage of the corresponding control value. ^***^p < 0.01, ^**^p < 0.01, ^*^p < 0.05.

To investigate whether there are regulators that modulate the expression of β-catenin protein, we analyzed SMSP (at 10^−7^ M) or L-733060 (at 10^−6^ M) -treated melanocytes for the gene expression levels of receptors, ligands, and many intracellular factors involved in the Wnt/β-catenin signaling pathway, such as Wnt3a, lipoprotein receptor-related protein 5/6 (LRP5/6), Frizzled, Dvl, Axin2, GSK3β, CK1α, F-box protein β-transduction repeat-containing protein (β-TrCP), TCF, and β-catenin. No significant changes were evident in the expression of these genes (Figure 3A). Next, we examined the gene expression levels of modulators that are known to block interactions between ligands and receptors in the Wnt/β-catenin signaling pathway. The qPCR study demonstrated that SMSP could impair the expression of several modulator genes including DKK1, WIF1, sFRP2, and sFRP5, while heightened that of DKK3. The expression of inhibitors DKK2, sFRP1, and sFRP4 was unchanged. L-733060 could affect DKK1, WIF1, sFRP2, and sFRP5, and increase DKK3 expression (Figure 3B). Interestingly, there was a marked increase or decrease in DKK1 gene expression. These results suggest that NK-1R selectively regulate modulators of Wnt/β-catenin signaling, including DKK1.

We then examined the expression levels of DKK1 protein in melanocytes treated with SMSP or L-733060. The western blot results were also in favor of the qPCR results, which showed that SMSP could weaken DKK1 protein expression level, but L-733060 heightened that (Figure 3C–3D).

**Figure 4:**
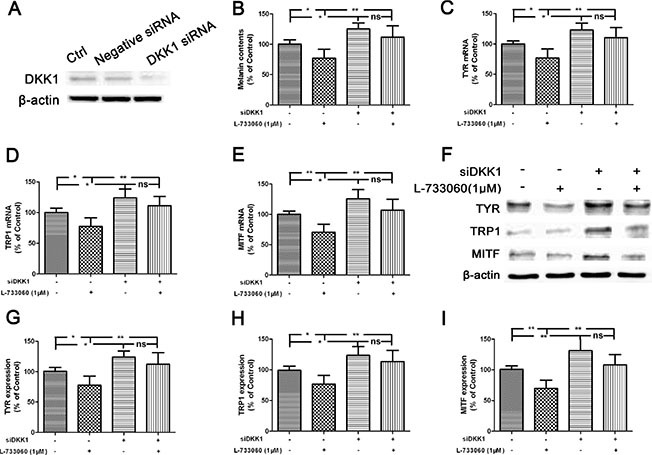
Inhibition of melanogenesis by L-733060 through the up-regulation of DKK1 in the human melanocytes (**A**) Melanocytes were transfected with Negative siRNA or DKK1 siRNA for 24 h, and then cells were harvested to detect DKK1 protein levels by Western blot. After treatment with SMSP for 24 h, the melanin content (**B**), the gene (**C**–**E**) and protein (**F**–**I**) expression of TYR, TRP1 and MITF were detected. Statistical results from the densitometric measurements after normalization against β-actin were calculated as the mean ± SD (*n* = 6). Values are expressed as a percentage of the corresponding control value. ^**^p < 0.01, ^*^p < 0.05.

**Figure 5:**
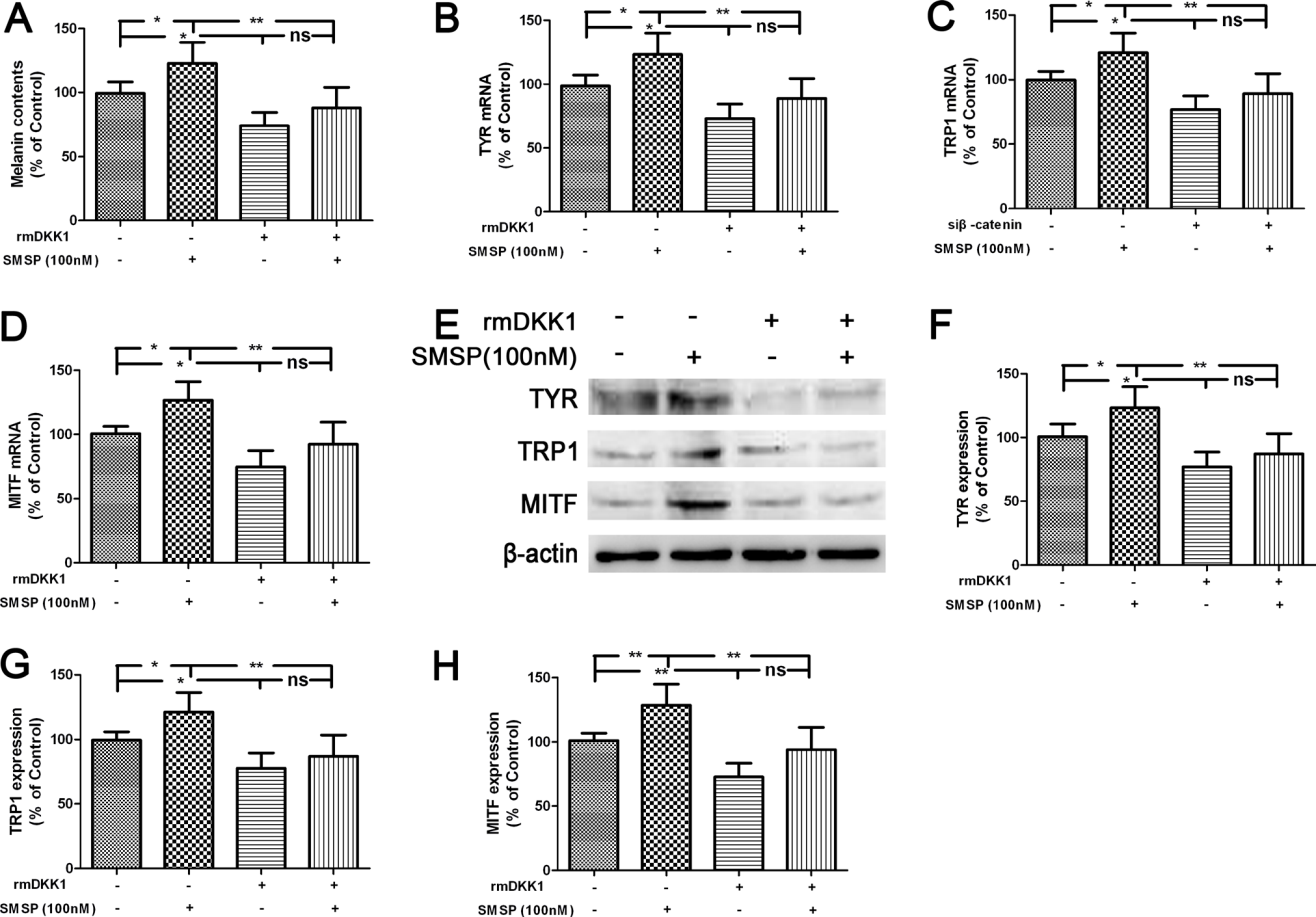
Promotion of melanogenesis by SMSP through the down-regulation of DKK1 in the human melanocytes Cells were treated with rmDKK1 (100 ng/ml) and the melanin content (**A**), the gene (**B**–**D**) and protein expression (**E**–**H**) of TYR, TRP1 and MITF were detected. Statistical results from the densitometric measurements after normalization against β-actin were calculated as the mean ± SD (*n* = 6). Values are expressed as a percentage of the corresponding control value. ^**^p < 0.01, ^*^p < 0.05.

To verify whether DKK1 participated in L-733060-regulated melanogenesis, cells were transfected with siDKK1. As shown in Figure 4A, DKK1 small interfering RNA (siRNA) significantly down-regulated the level of DKK1 expression in human melanocytes. The decreased expression of DKK1 attenuated L-733060-induced decrease of the melanin content and downregulation of melanogenesis-related genes and proteins including TYR, TRP1 and MITF gene and protein expression (Figure 4B–4I). To further confirm these observations, the exogeneous recombinant mouse (rm) DKK1 was applied. As expected, the increase of melanin content and upregulation of TYR, TRP1 and MITF expression were substantially curtailed after rmDKK1 treatment (Figure 5). These results indicated that NK-1R activation increased melanin synthesis through Wnt/β-catenin signaling by the inhibiting the Wnt inhibitor gene DKK1, suggesting that DKK1 is a critical factor for pigmentation inhibition of melanocytes.

### NK-1R activation promotes melanogenesis through Wnt/β-catenin signaling pathway in human melanocytes

**Figure 6:**
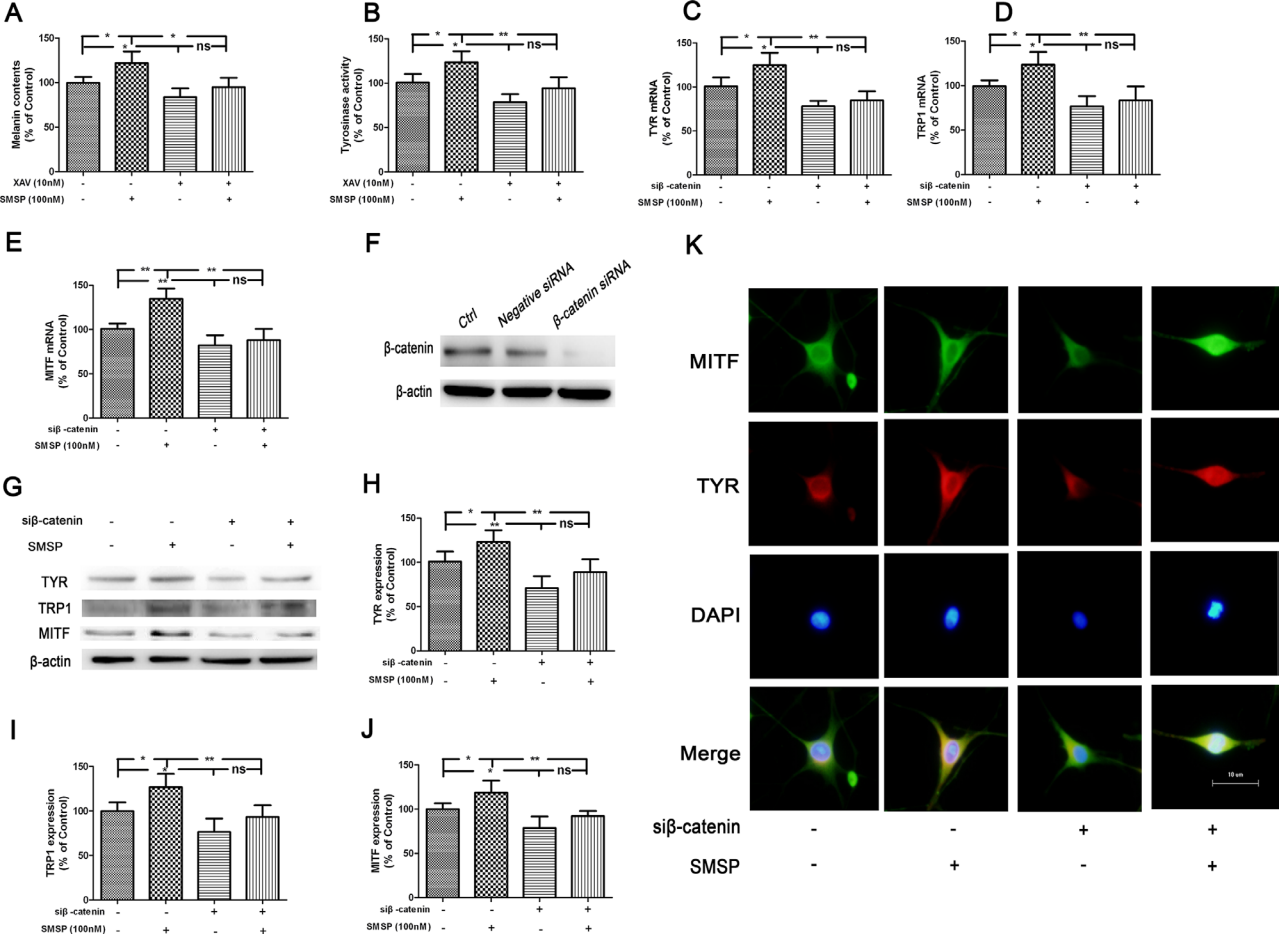
Promotion of melanogenesis by SMSP through the activation of Wnt/β-catenin signaling pathway in the human melanocytes After treatment with XAV, a Wnt/β-catenin inhibitor, the melanin content (**A**) and TYR activity (**B**) were assayed. After treatment with siβ-catenin, the mRNA levels of TYR, TRP1 and MITF were detected (**C**–**E**). (**F**) Melanocytes were transfected with Negative siRNA or β-catenin siRNA for 24 h, and then cells were harvested to detect β-catenin protein levels by Western blot. Melanocytes were transfected with siβ-catenin for 24 h. The transfected cells were treated with SMSP for 24 h and the expression of TYR, TRP1 and MITF protein were examined by Western blot (**G**–**J**) and immunofluorescence (**K**). Statistical results from the densitometric measurements after normalization against β-actin were calculated as the mean ± SD (*n* = 6). Values are expressed as a percentage of the corresponding control value. ^**^p < 0.01, ^*^p < 0.05.

To verify whether Wnt/β-catenin signaling participated in NK-1R-regulated melanogenesis, the inhibitor of XAV939, a Wnt/β-catenin inhibitor, was added 30 min before SMSP. As shown in Figure 6A and 6B, pretreatment with XAV939 abolished SMSP-induced elevation of melanin content and TYR activity. To further confirm these observations, cells were transfected with siβ-catenin. Consequently, β-catenin small interfering RNA (siRNA) significantly down-regulated the level of β-catenin expression in human melanocytes (Figure 6F). The decreased expression of β-catenin abolished SMSP-induced enhancement of the levels of melanogenesis-related genes and proteins including TYR, TRP2 and MITF expression (Figure 6C–6E and 6G–6J). In immunofluorescence assays, siβ-catenin-treated melanocytes was less positive for MITF and TYR compared with that in SMSP-treated cells (Figure 6K). These results verify that Wnt/β-catenin was involved in NK-1R-mediated melanogenesis *in vivo*.

### NK-1R inhibition suppresses melanin production and melanogenic protein expression in C57BL/6J mice

**Figure 7:**
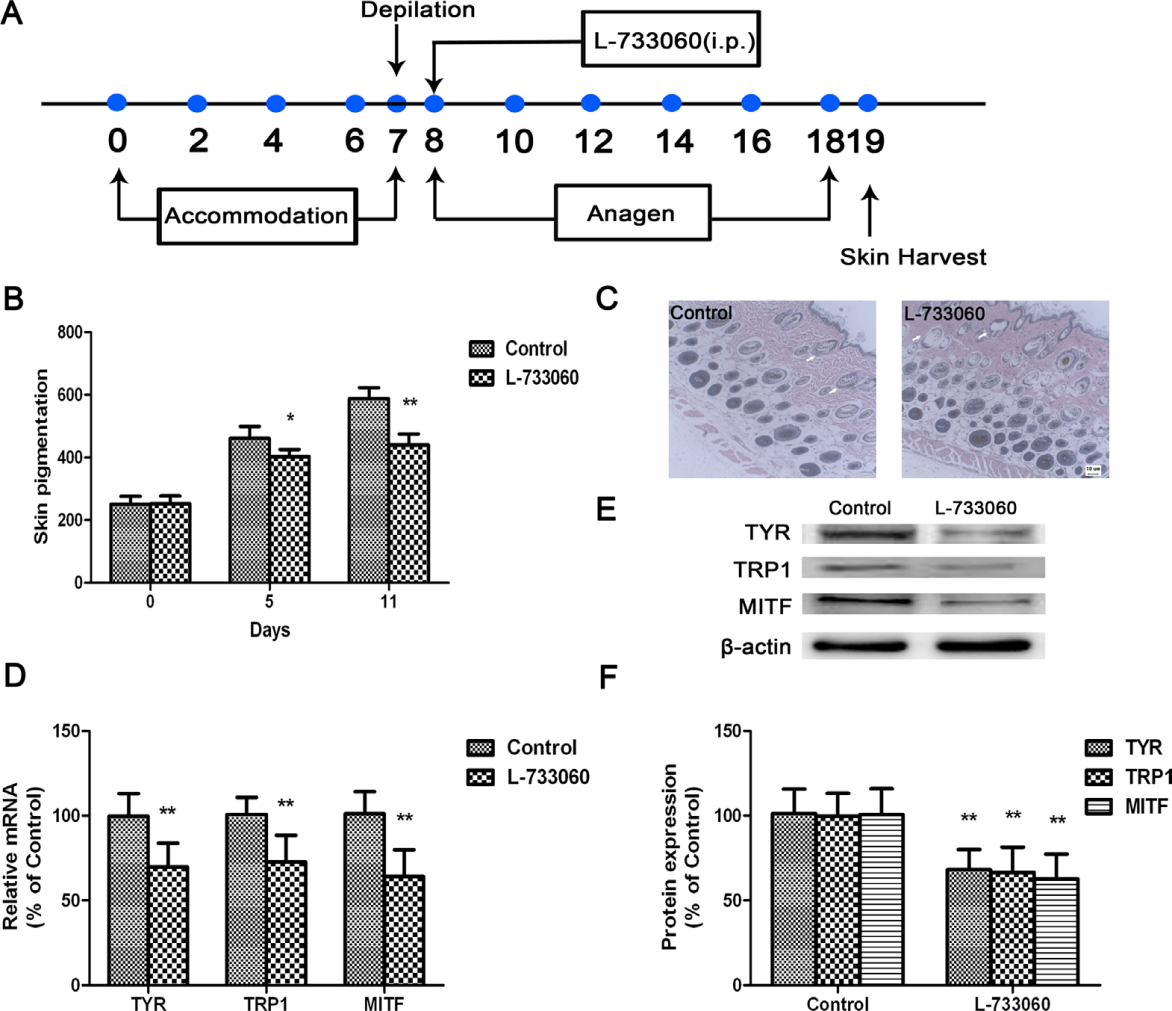
L-733060 inhibited melanin production in C56/BL6J mice (**A**) Representative scheme of experiments. (**B**) Measurement of melanin content by a Mexameter. (**C**) Skin sections were stained with hematoxylin and eosin and examined under light microscope. The mRNA and protein levels of TYR, TRP1 and MITF were detected by Real-time qRCR (**D**) and Western blot (**E**–**F**). Statistical results from the densitometric measurements after normalization against β-actin were calculated as the mean ± SD (*n* = 6). Values are expressed as a percentage of the corresponding control value. ^**^p < 0.01, ^*^p < 0.05.

To further examine whether NK-1R regulates melanogenesis *in vivo*, we applied L-733060 (10 mg/kg, i.p.) or saline to the C57BL/6J mice with shaved dorsal skin (Figure 7A). Firstly, to observe the dorsal skin of mice, colorimeter measurements and HE staining were taken. Compared with the Control group, L-733060 markedly decreased the pigmentation levels of mice (Figure 7B–7C).

Secondly, to investigate the expression of melanogenic proteins in L-733060-treated mice, the TYR, TRP1 and MITF in the dorsal skin was analyzed by qPCR. The gene expression of these molecules were down-regulated compared with that of Control group (Figure 7D). The results shown by western blot is consistent with that of qPCR (Figure 7E–7F).

### NK-1R inhibition suppresses melanogenesis through Wnt/β-catenin signaling pathway in C57BL/6J mice

**Figure 8:**
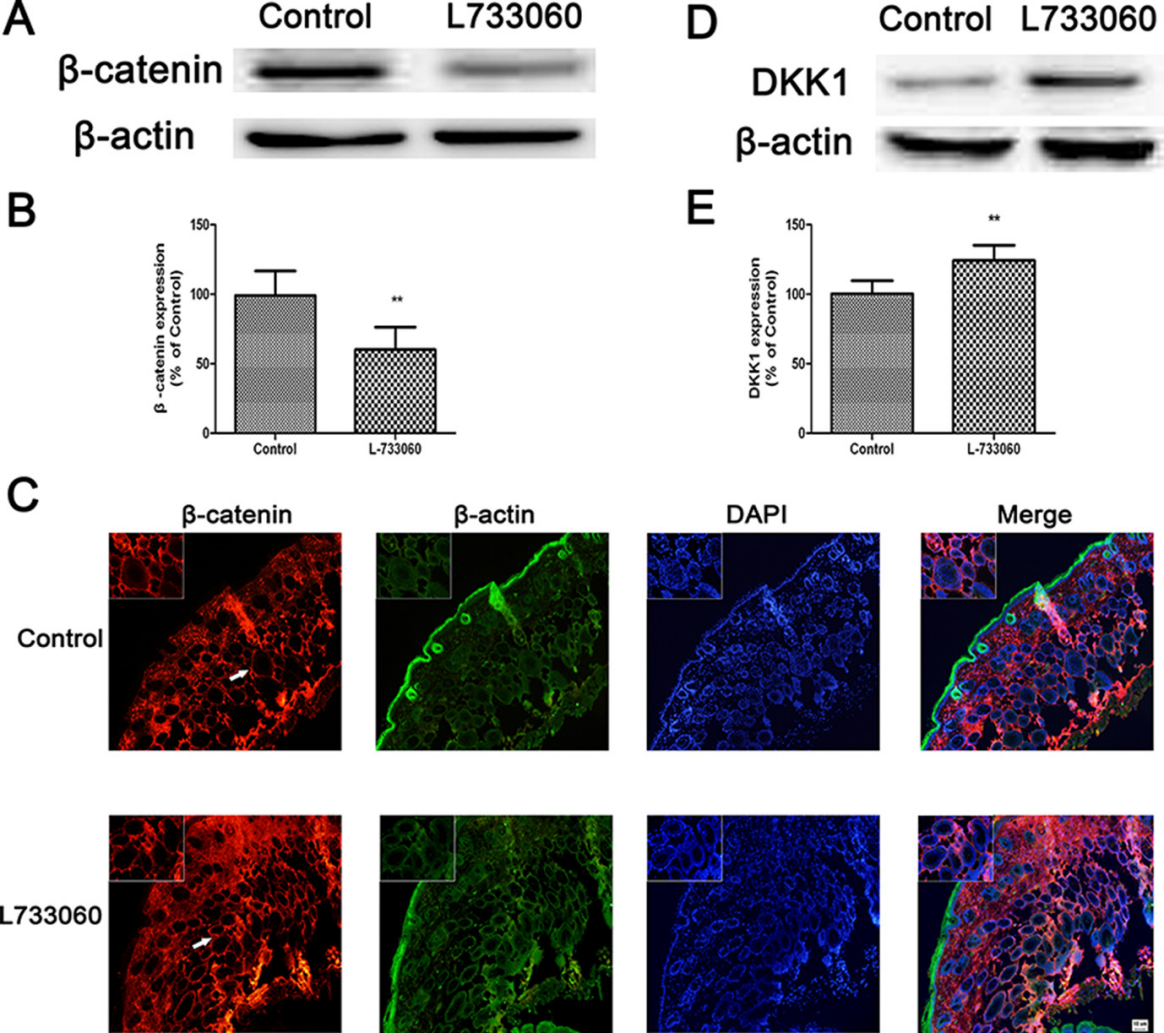
L-733060 decreased the expression of β-catenin in C56/BL6J mice The protein level of β-catenin by western blot (**A**–**B**) and immunofluorescence (**C**). (**D**–**E**) The DKK1 protein level was detected by western blot. Statistical results from the densitometric measurements after normalization against β-actin were calculated as the mean ± SD (*n* = 6). Values are expressed as a percentage of the corresponding control value. ^**^p < 0.01, ^*^p < 0.05.

Finally, to confirm whether the melanin inhibition in L-733060-treated dorsal skin is associated with Wnt/β-catenin signaling pathway, the dorsal skin of mice was analyzed by immunofluorescence using antibodies against β-catenin. L-733060-treated dorsal skin revealed a strong degradation of β-catenin in cytoplasm compared with that in non-treated dorsal skin (Figure 8A–8C). Besides, the expression of DKK1 was significantly increased in L-733060-treated dorsal skin tissues (Figure 8D–8E).

## DISCUSSION

NK-1R mediates several physiological and pathophysiological responses [31]. The pharmacological antagonists of NK-1R have been used so far for treating diverse conditions, such as mood disorders (depression, anxiety and stress), nausea associated with chemotherapy, rheumatoid arthritis, and inflammatory bowel disease [32, 33]. However, there is little literature reporting the role of NK-1R in melanogenesis. Previously, we demonstrated that NK-1R was expressed in the B16 melanoma cells and human primary melanocytes, as well as SP inhibited melanin production through the activation of NK-1R [22]. Because Wnt/β-catenin signaling is essential for melanin sythesis in melanocytes [34], strategies have been developed to regulate Wnt signaling for melanogenesis. On the basis of the above, we hypothesized that the cross talk between NK-1R and Wnt/β-catenin might be involved in melanogenesis.

To address this hypothesis, we firstly performed *in vitro* NK-1R stimulation (SMSP) and inhibition (L-733060) experiments to investigate the effects of NK-1R on the expression of β-catenin and GSK-3β. The results showed that SMSP increased the expression of β-catenin and p-GSK3β, while L-733060 decreased these markers. GSK-3β, a negative regulator in the Wnt/β-catenin signaling pathway, could activate the function of MITF through phosphorlyation at Ser 298. Activated GSK-3β induces phosphorlyation of the N-terminal Ser/Thr residues in β-catenin, which leads to the ubiquitination and degradation of β-catenin. To examine whether the expression level of β-catenin protein is directly regulated by GSK-3β, BIO, the GSK-3β inhibitor was applied before SMSP and the results demonstrated that pretreatment with BIO abolished SMSP-induced up-regulation of β-catenin protein expression. These findings suggest the positive regulation of NK-1R on Wnt/β-catenin signaling pathway in melanocytes.

Next, we analyzed the gene expression levels of Wnt inhibitors in SMSP or L-733060-treated cells by qPCR. We found that SMSP treament significantly suppressed Wnt inhibitors DKK1, WIF1, sFRP2, and sFRP5. On the contrary, L-733060 obviously activated these markers. In particular, the level of DKK1 mRNA and protein was robustly increased in L-733060-treated melanocytes *in vitro* and in C57BL/6J mice *in vivo*. DKK1 is an inhibitor of the canonical Wnt/β-catenin signaling [35], which interacts with LRP5/6 [36]. The observed increases in the expression of Wnt inhibitors DKK1, WIF1, sFRP2, and sFRP5 induced by L-733060 indicate that NK-1R inhibition may interfere with the interaction between transmembrane receptors Frizzled and LRP5/6 with the Wnt ligand.

To confirm the role of Wnt/β-catenin signaling in SMSP-induced melanogenesis, we treated the normal human mealocytes with Wnt/β-catenin inhibitor XAV939 or siβ-catenin. Results showed that Wnt/β-catenin inhibition by XAV939 blocked SMSP-induced elevation of melanin content and TYR activity. It is well known that skin color depends on melanogenesis of melanocytes in association with the melanogenic proteins, including TYR, TRP1 and Dct/TRP2 [37]. Microphthalmia-associated transcription factor (MITF) is a crucial regulator of melanocyte survival and the expression of melanogenic enzymes like enzymes like TYR and tyrosinase-related proteins [38, 39]. In the present study, we demonstrated that the up-regulation of TYR, TRP1 and MITF gene and protein expression were reversed after pretreatment with a β-catenin-specific siRNA. These findings indicate that NK-1R regulates melanogenesis through Wnt/β-catenin signaling pathway. Generally, the above findings reveal that NK-1R inhibition contribute to the disruption of Wnt/β-catenin through up-regulation of DKK1 expression.

To confirm the *in vitro* data showing the role of NK-1R in melanogenesis *in vitro*, we further investigate the effects of L-733060 on melanin synthesis in C57BL/6J mice *in vivo*. The results showed that L-733060 treatment decreased pigmentation level and TYR, TRP1 and MITF gene and protein expression in mice. Moreover, to verify whether Wnt/β-catenin is involved in the NK-1R-mediated melanogenesis *in vivo*, the expression of β-catenin and various Wnt inhibitors were then analyzed. It was found that L-733060 markedly down-regulated the expression of β-catenin protein at nuclear in the dorsal skin tissues of mice. Interestingly, NK-1R inhibition robustly increased the expression of a Wnt inhibitor, DKK1, which results in an obvious suppression of β-catenin protein expression.

Of note, when we draw the conclusion of this paper, we realized the contradicting data, previous reports demonstrate that activating NK-1R inhibits melanogenesis whereas this reports suggests it activates it via up-regulating Wnt/β-catenin signalling. In order to find a reasonable explanation, we analyzed in detail the reasons that may lead to inaccurate results. On the premise that the results are correct, we give the following explanation to this contradiction. (1) In our previous paper, we used the substance P (SP), and here we used [Sar^9^, Met (O_2_)^11^] substance P (SMSP). The formula of SP is C_63_H_98_N_18_O_13_S [Arg-Pro-Lys-Pro-Gln-Phe-Phe-Gly-Leu-Met-NH_2_], while that of SMSP is C_64_H_100_N_18_O_15_S [Arg-Pro-Lys-Pro-Gln-Gln-Phe-Phe-Sar-Leu-Met-(O_2_)-NH_2_]. Since their structures are not exactly the same, we speculate that there may exist the differences between their actions. Take the biological activity into consideration, SMSP is the potent selective NK-1R agonist. NK-1R has a high affinity for tachykinin family members, such as SP [40]. Endogenous tachykinins are not highly selective for any given receptor, and all can act on all three receptors under certain conditions such as receptor availability or at high peptide concentrations. For this reason SP activates not only NK-1R, but also NK-2R and NK-3R in a number of tissues [41]. In other word, the selectivity of synthetic SMSP may be superior to endogenic SP. (2) SP is one of the endogenous tachykinins, an important autocrine regulator with many biological effects. Under different stimulus, or different cell origins and types under the same stimulus, the levels of endogenous SP may be different. This further causes the discrepancy in pigmentation. Accordingly, we speculate that the differences of structures between SMSP and SP may lead to their different levels or even effects when they play a role in pigmentation. Nevertheless, the reason and mechanism of the differences remain to be investigated. (3) The G protein–coupled receptors, also known as seven-transmembrane receptors (7TMRs), represent by far the largest family of plasma membrane receptors and mediate a dizzying array of physiological functions. Three families of proteins mediate the function of the receptors: the heterotrimeric G proteins, the G protein–coupled receptor kinases (GRKs), and the β-arrestins. β-arrestins have emerged as remarkably versatile adaptor molecules that regulate receptor endocytosis [42]. Recent evidence shows that β-arrestins can also function to activate signaling cascades independently of G protein activation. There may be several active conformation of one GPCR, different ligands lead to different receptor conformation changes. Some receptor conformation is conducive to the binding with G protein, and other with β-arrestin. Now the β-arrestin-biased ligands are hot studying field. It has been reported that G protein receptor kinases (GRKs) phosphorylate SP-bound NK-1R to promote interaction with β-arrestins, which mediate NK-1R desensitization and endocytosis, terminate NK-1R signaling, and then rapid NK-1R recycling and resensitization [43]. NK-1R is a member of the Gq protein-coupled receptor family, after binding with SP, the NK-1R caused the activation of p38 and p42/44 MAPK, NF-κB and PKC, and thereafter to increase the production of PGE2 and the expression of COX-2 [44]. The differences between SMSP and SP, or rather the differences of bias on β-arrestin/G-protein, causing different signal pathways, may be one reason for the further differences on pigmentation.

**Figure 9:**
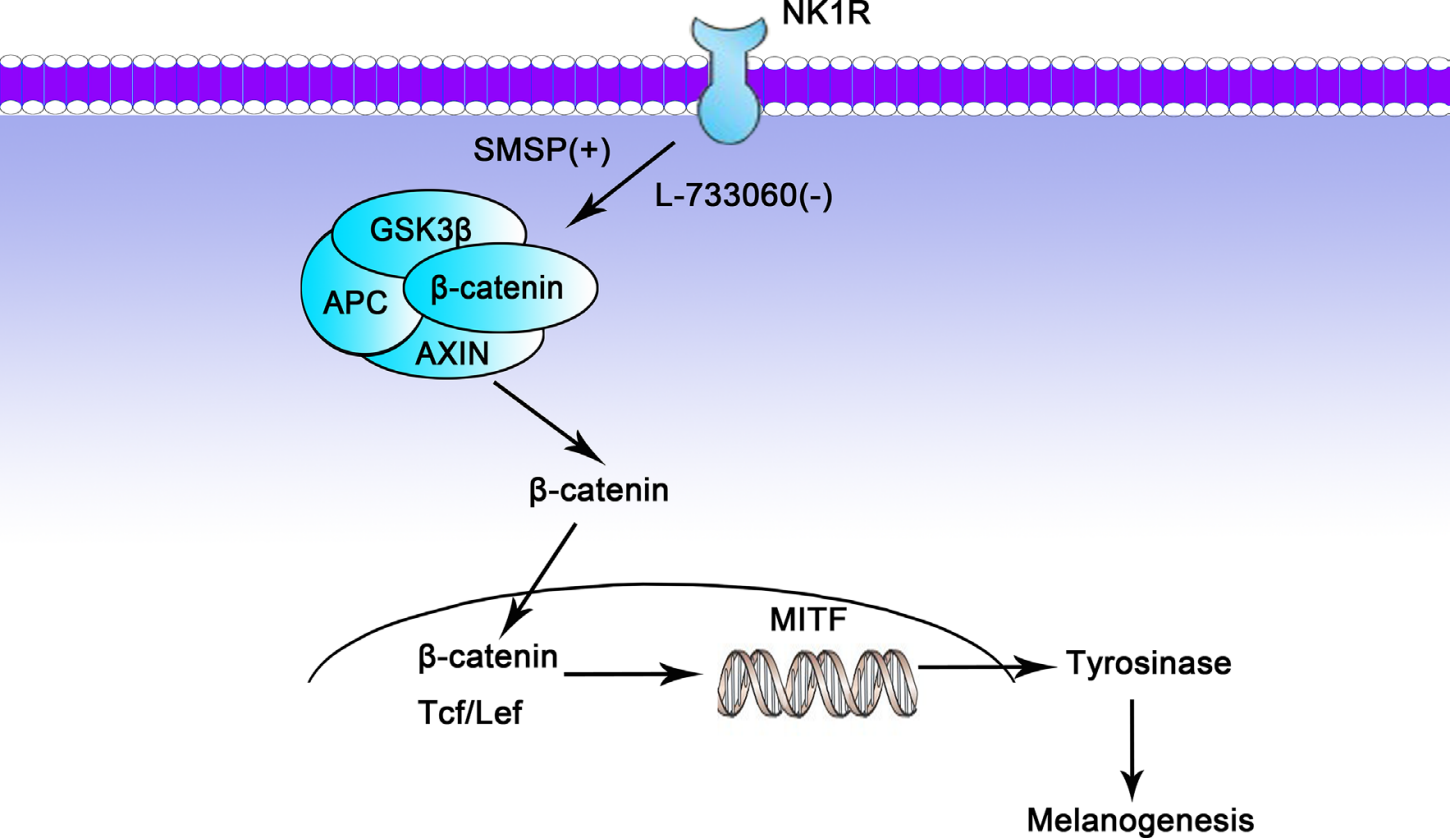
Potential mechanism by which NK-1R may regulate melanogenesis through Wnt/β-catenin signaling pathway SMSP: the NK-1R stimulator; L-733060: the NK-1R inhibitor; +: stimulate; −: inhibit.

In summary, the current study evaluated the relationship between NK-1R and Wnt/β-catenin in melanogenesis in human melanocytes *in vitro* and C57BL/6J mice *in vivo*. Our results suggest that NK-1R plays a positive role in melanogenesis through Wnt/β-catenin signaling pathway (Figure 9). The present findings provide important clues to understand the roles and mechanisms of NK-1R that is involved in melanogenesis, which suggests the potential value of NK-1R for skin pigmentation regulation. Despite the current results, more experiments are needed to further confirm the interaction between NK-1R and Wnt/β-catenin signaling pathway in melanogenesis. Following studies in our laboratory will focus on the related proteins of Wnt family, extending the administration time of NK-1R antagonist, using the NK-1R gene knockout mice and so on.

## MATERIALS AND METHODS

### Materials

Primary antibodies were purchased from Cell Signaling Technology (Beverly, MA, USA), Santa Cruz Biotechnology (Santa Cruz, CA, USA), or Abcam (Cambridge, UK). [Sar^9^, Met(O_2_)^11^] substance P (SMSP) and (2S,3S)-3-[(3,5-bis (Trifluoromethyl) phenyl) methoxy]-2-phenylpiperidine hydrochloride (L-733060) were purchased from Tocris (Bristol, UK). XAV939, 6-bromoindirubin-3-oxime (BIO) and other chemicals were purchased from Sigma-Aldrich (St Louis, MO, USA). The siRNA construct used was obtained as mismatched siRNA control (Negative Control, Santa Cruz Biotechnology, Santa Cruz, CA), siRNA against β-catenin (isβ-catenin, Cell Signaling Technology Co., Ltd, MA, USA). C57BL/6J mice were purchased from SLAC Laboratory Animal Co, Ltd (Shanghai, China). Animal care and experimental procedures were followed according to institutional guidelines and conformed to requirements of the authority for animal research conduct at the respective institutions.

### Cell culture and treatment

The studies on human material were approved by local ethic committee. Normal human foreskin-derived epidermal melanocytes (NHEM) were derived from young male adult foreskins (ethnic Han/aged 18 to 22 years) obtained at circumcision following standard protocol [45]. Briefly, foreskins were cut into strips and digested with 0.25% trypsin at 4°C for 20 h. Epidermis was separated from dermis. The NHEM suspension was filtered and cells were washed twice at 1,500 rpm for 5 min prior to resuspension in Medium 254 (containing the HMGS). NHEM were grown in a humidified atmosphere with 5% CO_2_ at 37°C. Through about two-week cell culture, we collected melanocytes by 0.25% trypsin (containing EDTA) at 37°C for about 30–50s. This time period was not enough for keratinocytes to be digested and the melanocytes were purified. Purity melanocytes of passage 2 to 5 can be used for the experiments, and we choose passage 4. The following all treatments were performed 3 times or more, we used one sourced melanocytes for single trial. In other words, 3 times of trials we used 3 different individual sourced melanocytes. SMSP or L-733060 was dissolved in distilled water to obtain the storing solution at 0.5 mM and 20 mM, respectively, and then diluted in medium to obtain experimental concentrations (SMSP,10^−5^M-10^−9^M; L-733060, 10^−4^M-10^−8^M).

### RNA interference

Normal human melanocytes were plated and grown in 60-mm culture dishes. After overnight, they were transiently transfected with 10–50 nM siRNA using lipofectamineTM 2000 (Invitrogen, CA, CA) based on the manufacturer's instruction. At 24 h after transfection, the cells were treated with SMSP for 24 h and examined by Western blot analysis.

### Melanin content and tyrosinase activity assay

For melanin content determination, cell pellets were solubilized in 1M NaOH. The absorbance at 490 nm was compared with a standard curve of synthetic melanin (Sigma) using Thermo Scientific Microplate Reader. For assay of tyrosinase activity, cells were solubilized with 1% SDS, 1% Tween-20 and 1 mM L-DOPA (Sigma) in PBS (pH 6.8). Following 90-min incubation at 37°C, the absorbance was measured at 490 nm.

### Animals and treatment

C57BL/6J mice were maintained in an animal facility with a 12-hour light/dark cycle. After a 1-week acclimation period, the mice were divided into two groups (*n* = 6 for each group): Control group and L-733060-treated group. Before we performed the experiment, depilation is conducted to induce a synchronization of the hair cycle stage in all mice (see below for method). L-733060 was dissolved/sonicated in 10 μl Tween 80, with their final volumes adjusted for i.p. injection (10 ml/kg) with 0.9% sterile saline. All mice received L-733060 (10 mg/kg) or saline on days 8 to 18 and were sacrificed 11 days after depilation.

### Synchronization of hair cycle by depilation-induced anagen induction

Anagen was experimentally induced by depilation, as previously published [46]. Briefly, on day 19 mice were anesthetized with an intramuscular injection of sodium pentobarbital (30 mg/kg, i.p.) Then, a wax/rosin mixture was applied to the dorsal skin of mice with all hair follicles in telogen, as evidenced by the pink back skin color. Peeling off the wax/rosin mixture removes all hair shafts and immediately induces homogeneous anagen development over the entire depilated back skin area of the mouse, thus inducing a highly synchronized anagen development. After full anagen development, the consecutive stages (catagen and telogen) then develop spontaneously in relatively homogeneous wave-like pattern, starting in the neck region [46].

### Measurement of pigmentation

The dorsal skin pigmentation of mice was measured with a Mexameter (MX18, Germany). The melanin index was automatically calculated from the intensities of absorbed and reflected light at 660 and 880 nm, respectively [47].

### Immunocytochemistry

Skin biopsies were fixed in 4% formaldehyde. Paraffin-embedded sections were cut to 5 μm thickness and the skin tissue sections were stained with hematoxylin and eosin (HE).

Immunocytochemistry procedures were performed as previously reported (Ping et al., 2012). Briefly, Cells grown on coverslips and Cryosections (5 μm) were fixed in 4% paraformaldehyde for 20 min and then permeabilized with 0.01% Triton X-100 in PBS for 10 min, blocked for 30 min with PBS containing 10% normal horse serum, and incubated overnight at 4°C with anti- MITF (1:200), anti-tyrosinase (1:200), or anti-β-catenin (1:100) antibodies. Cells and sections were then incubated for 60 min with Alexa Fluor 488-conjugated donkey anti-goat IgG and Alexa Fluor 594-conjugated donkey anti-rabbit IgG. Finally, nuclei were counterstained with DAPI. The staining was visualized using an Olympus IX71 microscope.

### Western blot analysis

The Western blot analysis was performed as described previously (Ping et al. 2012). Briefly, dorsal skin homogenate supernatant or cells lysed in cold RIPA buffer (pH 7.4) containing protease inhibitor cocktail. The total protein concentration was measured and adjusted to equal concentrations across different samples. The protein was separated on a 10% SDS-PAGE gel and transferred onto a polyvinylidene difluoride (PVDF) membrane (Millipore, MA, USA). The PVDF membranes were incubated with the indicated primary antibodies overnight at 4°C, and then incubated with the secondary antibodies conjugated to horse-radish peroxidase. The proteins were visualized by a Keygen ECL system (Kaiji, Nanjing, China) and scanned with a Clinx Chemi-Scope chemiluminescence imaging system (Gel Catcher 2850, China). The relative optical densities of the specific proteins were determined by a ChemiScope analysis program.

### RT-PCR

Total RNA was isolated from the cells using TRIzol reagent (Invitrogen, CA, USA). All reactions were performed with a final reaction volume of 20 μl containing 10 μl of iTaq Universal SYBR Green Supermix(Bio-Rad), 500 nM for each primer and 1 μl of RT product. Real-time PCR assays were carried out under the following conditions: An initial preheating step of 10 min at 95°C, then a touch-down procedure, consisting of 10 s at 95°C, annealing for 5 s at temperatures decreasing from 63 to 59°C, and ending with an extension step at 72°C for 10 s. A total of 45 cycles were performed, followed by melting curve program (60–95°C with a heating rate of 0.1°C per second and a continuous fluorescence measurement), and finally a cooling step to 40°C. PCR primers are listed in Supplementary Table S1.

### Statistical analyses

All data are expressed as mean ± SD. Statistically significant differences were calculated using independent and paired Student's paired *t*-test on unpaired and paired samples.

## SUPPLEMENTARY MATERIALS


