# Supplementary material for: Neurokinin-1 receptor is a novel positive regulator of Wnt/β-catenin signaling in melanogenesis
Source: Oncotarget. 2016 Nov 8;7(49):81268–80. doi: 10.18632/oncotarget.13222 (PMC5348391; doi:10.18632/oncotarget.13222)
Supplement: Supplementary file 1 [file oncotarget-07-81268-s001.pdf]

Neurokinin-1 receptor is a novel positive regulator of Wnt/β-catenin signaling in melanogenesis

Supplementary Materials

Supplementary Table S1: Quantitative RT-PCR primers

Target	upstream	downstream
β-catenin	GTGCGCATGGAGGAGATAGTAG	GAATGGTATTGAGTCCTCGGATT
LRP5/6	GCAAACTCAGTCGCAAATCA	CTTTCTCGGGGTTTACCACA
Fzd	CAAGGTTTACGGGCTCATGT	CAAACTTGTCGTTGCACACC
Dvl	TATGTCTTCGGGGACCTCAG	CGAAGAAAGCTCGTGGTAGG
Axin2	GGGGGAAAACACAGCTTACA	ACTGGGTCGCTTCTCTTGAA
GSK3β	CAGTGGTGTGGATCAGTTGG	CAATTGCCTCTGGTGGAGTT
CK1α	GACAACAGGACAAGGCAACA	CCACGGCAGACTGGTTCTAT
β-TrCP	TCGTTTCTGCCTCTGGAGAT	CAGGCATGCTCCACACTCTA
TCF	CGCTCTGCAGCTCTCACTCT	CCTGTCTCTGAGATTCTTGTTGCTGCT
DKK1	GCCTCCGATCATCAGACTGT	GCAGGTGTGGAGCCTAGAAG
DKK2	CTGGTACCCGCTGCAATAAT	GTAGGCATGGGTCTCCTTCA
DKK3	ACCAGAGTGGACAGGTGGTC	CTCACTGTCTCGGGTGCATA
sFRP1	GCTCAACAAGAACTGCCACA	CTCGGGGAACTTGTCACATT
sFRP2	CCTGAAGAAATCCGTGCTGT	GGAGATGCGCTTGAACTCTC
sFRP4	CTGGCAACATACCTGAGCAA	ATCATCCTTGAACGCCACTC
sFRP5	TGGAGCCCAGAAGAAGAAGA	TTCTTGTCCCAGCGGTAGAC
WIF1	GAGTGTCCGGATGGGTTCTA	TGGTTGAGCAGTTTGCTTTG
Tyrosinase	GGCCAGCTTTCAGGCAGAGGT	TGGTGCTTCATGGGCAATC
Dct/TRP2	GGATGACCGTGAGCAATGGCC	CGGTTGTGACCAATGGGTGCC
TRP1	GCTGCAGGAGCCTTCTTTCTC	AAGACGCTGCACTGCTGGTCT
MITF	TACAGTCACTACCAGGTGCAG	CCATCAAGCCCAAAATTTCTT
